# Effects of myeloperoxidase on inflammatory responses with hypoxia in *Citrobacter rodentium*‐infectious mice

**DOI:** 10.1002/iid3.1157

**Published:** 2024-02-02

**Authors:** Xiang Gao, Yu Zhang, Qinfang Zhu, Ying Han, Ruhan Jia, Wei Zhang

**Affiliations:** ^1^ Department of Basic Medical Sciences, Medical College Qinghai University Xining Qinghai China; ^2^ Research Centre for High Altitude Medicine, Research Centre for High Altitude Medicine Qinghai University Xining Qinghai China; ^3^ The Key Laboratory of High‐Altitude Medical Application of Qinghai Province Xining Qinghai China

**Keywords:** colitis, hypoxia, inflammatory responses, MPO

## Abstract

**Purpose:**

Myeloperoxidase (MPO) has been identified as a mediator in various inflammatory diseases. Bacterial infection of the intestine and hypoxia can both lead to inflammatory responses, but the role of MPO in these phenomena remains unclear.

**Methods:**

By building the MPO^‐/‐^ mice, we evaluated relevant inflammatory factors and tissue damage in mice with intestinal *Citrobacter rodentium* infection and hypoxia. The body weight and excreted microorganisms were monitored. Intestinal tissues were collected 7 days after bacterial infection under hypoxia to undergo haematoxylin‐eosin staining and assess the degree of pathological damage. ELISA assays were performed to quantify the serum levels of TNF‐α, IFN‐γ, IL‐6, and IL‐1β inflammatory cytokines. PCR, WB, and IF assays were conducted to determine the expression of chemokines MCP1, MIP2, and KC in the colon and spleen.

**Results:**

The *C. rodentium* infection and hypoxia caused weight loss, intestinal colitis, and splenic inflammatory cells active proliferation in wild‐type mice. MPO deficiency alleviated this phenomenon. MPO^‐/‐^ mice also displayed a significant decline in bacteria clearing ability. The level of TNF‐α in the serum and spleen was both lower in MPO^‐/‐^ hypoxia *C. rodentium*‐infected mice than that in wild‐type mice. The chemokines expression levels of MIP2, KC, and MCP1 in the spleen and colon of each bacterial infected group were significantly increased (*p* < .05), while in hypoxia, the factors in the spleen and colon were decreased (*p* < .05). MPO deficiency was found to lower the levels of these chemokines compared with wild‐type mice.

**Conclusion:**

MPO plays an important role of the inflammatory responses in infectious enteritis and hypoxia in mice, and the loss of MPO may greatly reduce the body's inflammatory responses to fight diseases.

## Graphical abstract

We examined the inflammatory response of MPO^‐/‐^ mice with intestinal bacterial infection merely, hypoxia merely, and bacterial infection accompanied with hypoxia than those in wild‐type mice to explore the role of MPO in innate immunity.

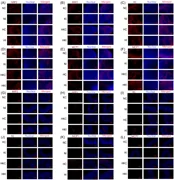

## 1 INTRODUCTION

Myeloperoxidase (MPO) is an important member of the haem peroxidase superfamily.^1^ It is found in primary azurophilic granules of neutrophils. In the presence of H_2_O_2_ and a halide—chloride or bromide, MPO can catalyzes the formation of reactive oxygen intermediates including hypochlorous (HOCl), and hypothiocyanous acids, respectively. Upon activation of neutrophils in peripheral blood and tissues, MPO is released into both the phagolysosomal compartment and the extracellular environment and then affects the aggregation of inflammatory cells under antigen stimulation.^2^, ^3^ Studies have shown that MPO not only plays a role in the process of killing pathogenic bacteria but also promotes the intensification of the inflammatory response and causes tissue damage.^4^ MPO is part of the innate immune system that helps phagocytes fight against invading microorganisms and can significantly increase its activities in the pathological process of neurological diseases, tumors, rheumatoid diseases, kidney damage, diabetes, and other diseases which participate in the initiation and progression of inflammation.^5^ To define the in vivo role of MPO in host defense, MPO‐knockout (MPO^‐/‐^) mice were created by two independent research groups and have been extensively studied for their susceptibility to infections. Mutant mice exhibit increased susceptibility to infection with *Candida albicans* and *Klebsiella pneumoniae* compared with infected wild‐type mice.^6^ Chami et al. pointed out that neutrophil‐myeloperoxidase (MPO) is increased in the mucosa of patients with IBD. MPO activity is an indicator commonly used to monitor IBD activity.^7^ However, the mechanisms of MPO in regulating intestinal immune responses during infectious colitis and the mice responses under hypoxia remain unclear. Here, we examined the inflammatory response of MPO^‐/‐^ mice with intestinal bacterial infection only, hypoxia merely, and bacterial infection accompanied with hypoxia to wild‐type mice to explore the role of MPO in innate immunity.

## 2 MATERIALS AND METHODS

### 2.1 The *Citrobacter rodentium* infection and hypoxia mice model

Sixty‐four (32 WT and 32 MPO^‐/‐^) C57BL/6 mice (SPF grade, 8‐week olds, female, 18–20 g) in total were purchased from Hu'nan SJA Laboratory Animal Co., Ltd. (WT mice) and Cyagen Biosciences (Suzhou) Inc. (MPO^‐/‐^ mice). The mice were kept in a specific‐pathogen‐free (SPF) facility with the Individual Ventilated Cage Animal Experiment System (H6, Su Hang Technology Equipment Co., Ltd.) with autoclaved water and food. Animal care was provided under protocols endorsed by the Institutional Animal Care and Utilize Committee at the Medical College of Qinghai University. All animal experiments comply with the ARRIVE guidelines. *Citrobacter* colitis was induced by oral feeding with *C. rodentium* (strain DBS100, ATCC number 51459, 5 × 10^8^ CFU/mouse). The hypoxia environment was made with a hypobaric chamber (Fukang Air Purification Equipment Engineering Co., Ltd.) at 5000 m (atmospheric pressure was 405 mmHg, PO_2_ was 84.7 mmHg) with IVC to keep exposure hypoxia air until the end of the experiment.^8^ Mice were randomly divided into eight groups with simple randomization method: WT normoxia control group (NC, *n* = 8), MPO^‐/‐^ normoxia control group (KC, *n* = 8), WT *C. rodentium* infection group (NI, *n* = 8), MPO^‐/‐^ *C. rodentium* infection group (KI，*n* = 8), WT hypoxia control group (HC, *n* = 8), MPO^‐/‐^ hypoxia control group (HKC, *n* = 8), WT hypoxia *C. rodentium*‐infected group (HI, *n* = 8) and MPO^‐/‐^ hypoxia *C. rodentium*‐infected group (HKI, *n* = 8). Eight mice were kept in one cage. Only feeder was aware of the group allocation at the different stages of the experiment.

### 2.2 Body weight and fecal bacterial output measurement

Body weight changes of the mice in each group were monitored every day from the first day of *C. rodentium* infection. The number of *C. rodentium* in each gram of feces (CFU/g) was calculated and continuously recorded for 7 days. At the same time, the feces of mice in the infected groups were collected and cultured on selective MacConkey agar (M8560; Solarbio). Bacterial colonies were then counted after culturing at 37°C.

### 2.3 Haematoxylin‐eosin (HE) staining

At necropsy, colonic tissues were collected, immersed in 4% paraformaldehyde (P110; Solarbio), and stored for 48 h at 4°C. Then, 5 μm sections were cut on a Leica RM2016 Microtome (Leica Biosystem) and stained with H&E. The colon and spleen tissues were fixed at 40 g/L in polyformaldehyde for 12 h. After dehydration, the tissue was turned transparent with xylene and washed with phosphate buffered saline (PBS). The sample was embedded in paraffin, and the tissue slice was stained. Colon lesions and inflammatory infiltration of spleen tissue were observed under an optical microscope. Histological scoring was based on two parameters as indicated in Table 1. The total colon pathology score equaled the inflammatory cell score plus the tissue damage score.

**Table 1:** Scores of intestinal tract inflammations.

Inflammatory cells	Score 1	Damage	Score 2
Normal	0	Normal	0
Scattered	1	Minimal inflammation and colonic crypt hyperplasia	1
Increased	2	Mild colonic crypt hyperplasia with or without focal invasion of the epithelium	2
Confluence	3	Obvious colonic crypt hyperplasia, invasion of the epithelium, and goblet cell depletion	3
Transmural extension	4	Extensive mucosal damage and extension to deeper structures of the bowel wall	4

### 2.4 ELISA

Mice were killed after hypoxic exposure for 7 days. On the 8th day, after blood collection from the mouse orbital vein, the mice were euthanized by cervical dislocation. The colon and spleen of the mice in each group were removed by surgery. After cutting the specimen, weigh it. Add a certain amount of PBS at a pH of 7.4. Quickly freeze and store in liquid nitrogen for later use. The specimen remains at a temperature of 2‐8°C after melting. Add a certain amount of PBS (pH 7.4) and homogenize the specimen thoroughly using hand or a homogenizer. Centrifuge for 20 min (2000–3000 rpm). Carefully collect the supernatant. After repackaging, one portion is to be tested, and the rest is to be frozen for later use. The blood samples were allowed to stand at room temperature for 1 h and processed by centrifugation at 3500 r/min for 15 min, and serum was collected and stored at −80°C. Double antibody sandwich ELISA kits were used to determine the levels of TNF‐α (JM‐02415M1; Jing Mei Biotechnology), IL‐1β (JM‐02465M1; Jing Mei Biotechnology), IFN‐γ (JM‐02446M1; Jing Mei Biotechnology) and IL‐6 (JM‐02323M1; Jing Mei Biotechnology) in serum samples and spleen lymphoid specimens. A total of 100 μL of serum was added to the packaged microwell plate and incubated at 37°C for 90 min. After discarding the supernatant and washing the plate, 100 μL of biotinylated cytokine‐specific antibody was added and incubated at 37°C for 60 min. Then, 100 μL of pro‐subsidin HRP was added and incubated at 37°C for 30 min. The substrate TMB was developed by placing the plate at room temperature for 15–20 min. Finally, the stop solution was added, the absorbance was measured at a wavelength of 450 nm by a microplate spectrophotometer. Each sample has three multiple pores. The results were analyzed by ELISA kits (ImmunoAssays SA).

### 2.5 Quantitative real‐time polymerase chain reaction (qRT‐PCR) analysis

Total RNA was extracted with a two‐step isocyanate method. The total RNA concentration was measured by an ultraviolet spectrometer, and the OD260/280 value ranged from 1.8 to 2.0. 5 μg of total RNA was used for reverse transcription to synthesize cDNA, and 3 μg of cDNA was used for PCR amplification. β‐Actin was regarded as an internal reference. Primers for β‐actin, TNF‐α, IFN‐γ, IL‐1β, MCP1, MIP2, and KC were synthesized by Primer Express. The PCR conditions included predenaturation at 94°C for 2 min, denaturation at 94°C for 30 s, annealing at 61°C for 45 s, and extension at 72°C for 1 min. The cycle was repeated 36 times, and each cycle included a reduction in annealing temperature by 0.3°C, with a final extension of 6 min at 72°C. The sequences of the primers used are listed in Table 2.

**Table 2:** Primers used in this study.

Primer	Sequence (5′–3′)
TNF‐α	Sense primer: GTGGAACTGGCAGAAGAGGC
	Antisense primer: CACAAGCAGGAATGAGAAGAGG
IL‐1β	Sense primer: TTGACCTGGGCTGTCCTGAT
	Antisense primer: TGAGTGATACTGCCTGCCTGAA
IFN‐γ	Sense primer: AAGTGGCATAGATGTGGAAGAAAA
	Antisense primer: GTTGCTGATGGCCTGATTGTC
MCP1/CCL2	Sense primer: TGCATCTGCCCTAAGGTCTTC
	Antisense primer: AGTGCTTGAGGTGGTTGTGGA
MIP2/CXCL2	Sense primer: CACCAACCACCAGGCTACAG
	Antisense primer: GCTTCAGGGTCAAGGCAAACT
KC/CXCL1	Sense primer: ACCCAAACCGAAGTCATAGC
	Antisense primer: ACAGGTGCCATCAGAGCAGT
GAPDH	Sense primer: CTCCACTCACGGCAAATTCA
	Antisense primer: ATACTCAGCACCGGCCTCAC

### 2.6 Western blot assay

The tissue was placed in a precooled homogenizer, and a lysis solution containing a protease and phosphatase inhibitor was added. After lysis for 15 min, the sample was centrifuged and stored at −80°C. The protein concentration was determined according to BCA. SDS–PAGE electrophoresis (100 g/L) was carried out after protein denaturation. The proteins were electrophoresed to a PVDF membrane, which was blocked with BSA reagent for 1 h. Primary antibodies (anti‐MCP1 [bs‐1955R; Bioss Antibodies], anti‐MIP2 [bs‐1162R; Bioss Antibodies], and anti‐KC [AB206411; Abcam], 1:1000) were added and incubated at 4°C overnight, and the membranes were washed five times with TBST. Then, an HRP‐labeled secondary antibody was added and incubated at room temperature for 1 h. The sample was developed with a Pierce ECL chemiluminescent kit.

### 2.7 Immunofluorescence assay

The colon and spleen tissue slices were blocked with goat serum for 30 min in each group. The goat serum was discarded, and the sample was washed twice with PBS and wiped with water‐absorbing paper. Fifty microliters of diluted antibody (1:500) was added to the sample, and PBS was added to the negative control group instead. After washing three times, fluorescent secondary (1:2000) was added, and the sample was incubated in the dark at room temperature for 2 h. Washing twice with PBS, adding an antiquenching agent dropwise, the sample was observed, and images were acquired under a fluorescence microscope.

### 2.8 Statistical analysis

Statistical analysis was performed using SPSS 27.0 software. The measurement data are expressed as $\bar{x}$ ± *S*, and multigroup comparisons were undertaken by single‐factor variance analysis or nonparametric statistics (rank sum test). The validation test was performed by the Levene test. When the variance was quarriable, the SNK test was used, and the multigroup comparison was chosen when it was different. *p* < .05 indicated that the difference was statistically significant.

## 3 RESULTS

### 3.1 Weight loss of mice was alleviated in MPO^‐/‐^ mice under bacterial infection and hypoxia exposure

**Figure 1:**
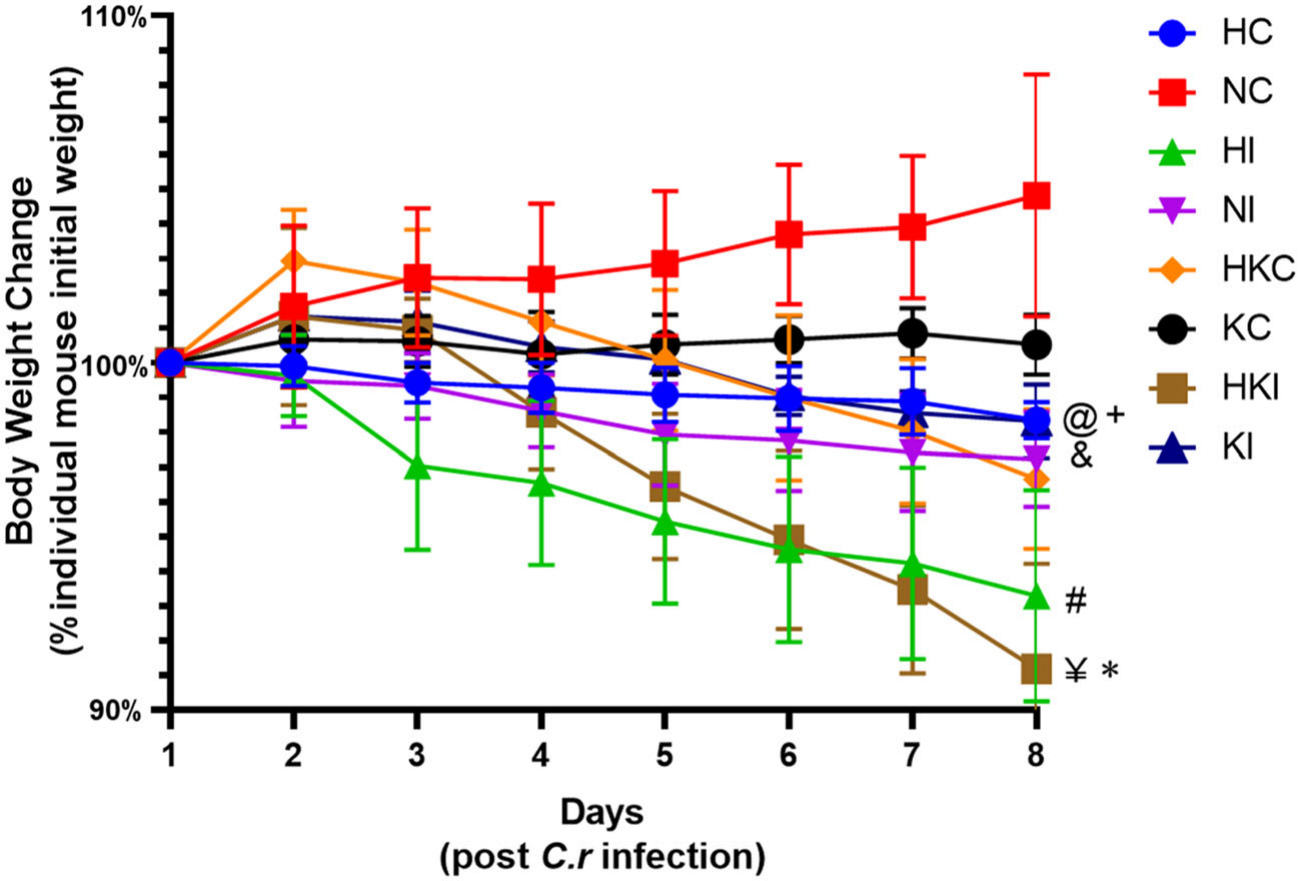
Changes in the body weight of mice in each group. The WT normoxia control group (NC), MPO^‐/‐^ normoxia control group (KC), WT *Citrobacter rodentium* infection group (NI), MPO^‐/‐^ *C. rodentium* infection group (KI), WT hypoxia control group (HC), MPO^‐/‐^ hypoxia control group (HKC), WT hypoxia C. *rodentium*‐infected group (HI) and MPO^‐/‐^ hypoxia *C. rodentium*‐infected group (HKI).

After 2 days of the experiment, the weight of mice in the infection groups decreased significantly compared with Day 0, (Figure 1). On the 4th day, the weight of the mice in the NI group showed an upwards trend, and that of the mice in the KI group decreased significantly. Compared with the control group, the infection groups showed a significant change in the body weight of the mice, and the body weight decreased significantly (*p* < .05), indicating that the infection caused the mice to lose weight; the HI group was compared with the NI and KI groups. The weight changes in KI and NI groups were relatively small, while the weight loss in HI group mice was more significant. The MPO‐H2O2‐halide system plays a crucial role in the rapid bactericidal effect of neutrophils. The vitro studies have shown that compared with normal neutrophils, MPO deficient neutrophils can lead to a weakened killing effect of the body against bacteria and fungi, and the degree of weakening varies with different pathogens.^9^ Due to the complete function of the MPO‐H_2_O_2_‐halide system in the HI and NI groups of mice, severe intestinal inflammation and significant weight loss were observed in the HI and NI groups. Compared with that of mice in the HKI group, there was a difference in body weight change (*p* < .05), and the weight loss of mice in the HI and HKI groups was more obvious, indicating that the hypoxic environment aggravated the weight loss of mice after infection. HI group lost weight more than HKI group before 6 days, we suspect that the effects of hypoxia on the body have a cumulative effect over time and take some time to show up. Comparing the MPO^‐/‐^ and WT mice, the weight of the WT mice decreased significantly (*p* < .05).

### 3.2 The lack of MPO expression increased greatly bacterial output and aggravated the shortening of the colon in mice with bacterial infection and hypoxia exposure

**Figure 2:**
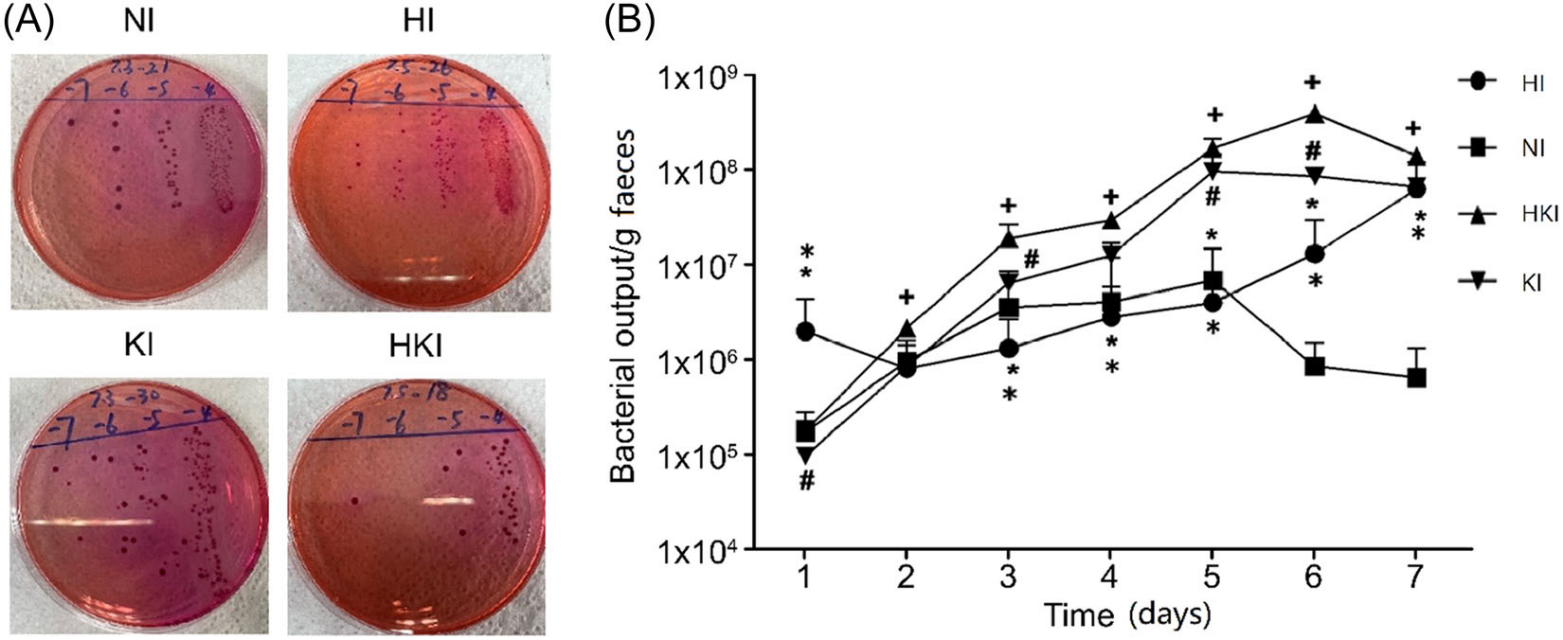
(A) Twenty‐four hours results of mouse fecal smear culture. (B) The number of bacteria excreted by mice in each group. The WT *Citrobacter rodentium*‐infection group (NI), MPO^‐/‐^ *C. rodentium*‐infection group (KI), WT hypoxia C. *rodentium*‐infected group (HI), and MPO^‐/‐^ hypoxia *C. rodentium*‐infected group (HKI).

**Figure 3:**
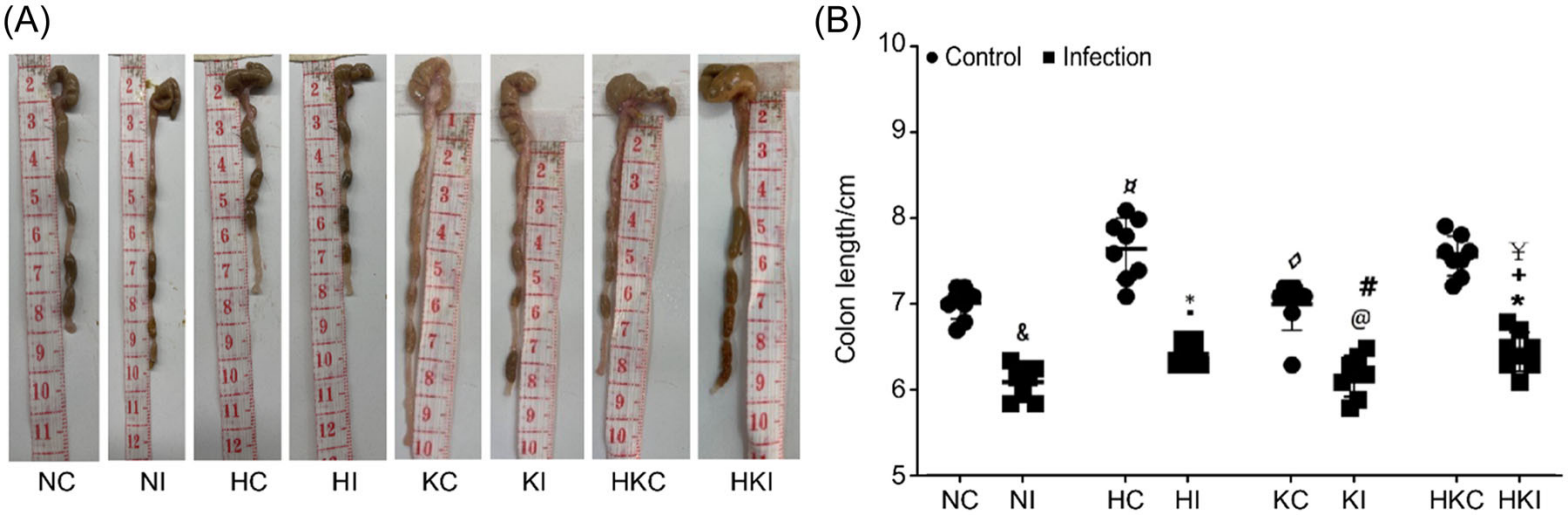
(A) Photographs of the colons of mice in each group. (B) Changes in the colon length of mice in each group. The WT normoxia control group (NC), MPO^‐/‐^ normoxia control group (KC), WT *Citrobacter rodentium* infection group (NI), MPO^‐/‐^ *C. rodentium* infection group (KI), WT hypoxia control group (HC), MPO^‐/‐^ hypoxia control group (HKC), WT hypoxia C. *rodentium*‐infected group (HI) and MPO^‐/‐^ hypoxia *C. rodentium*‐infected group (HKI).

The bacterial colonies in the feces of the mice were enumerated on the following day (Figure 2A). An increase in excretion indicated an increase in the severity of inflammation. Comparing the WT and MPO^‐/‐^ groups, the number of bacteria excreted in the MPO^‐/‐^ groups increased significantly. At the same time, we also found that compared with the HI group, the NI group had a significant decrease in the number of bacteria excreted, while the HI group had a continuous increase in the number of bacteria excreted. This indicates that the hypoxic environment at high altitude can inhibit the intestinal immune function of mice, leading to increased bacterial infection and increased bacterial excretion (Figure 2B). The total number of bacteria excreted in the HKI groups was the largest. We found that the colon lengths of the mice in the infected groups were significantly shorter (Figure 3A,B) after the mice were infected with *C. rodentium*, indicating that after the mice were infected, the colonic inflammation in the mice was aggravated as a result of colon shortening. Compared with that of the HKI group, the colon shortening of the KI group was more obvious, and there was a significant difference (*p* < .05), indicating that the colon shortening of the MPO^‐/‐^ mice in the hypoxic environment was not as obvious as that under normoxia. Comparing the NC with HC groups and the KC and HKC groups, we found that hypoxic exposure could significantly increase the colon length in mice (*p* < .05). The average length of each group is NC 7.01 cm, NI 6.05 cm, HC 8.75 cm, HI 6.43 cm, KC 7.00 cm, KI 5.78 cm, HKC 8.35 cm, HKI 6.45 cm. There were significant differences between the KI and NI groups and the HKI and HI groups (*p* < .05), which indicated that the deletion of the MPO gene could aggravate the shortening of the colon in mice.

### 3.3 Colitis and inflammatory cell infiltration in spleen was alleviative in MPO^‐/‐^ mice

**Figure 4:**
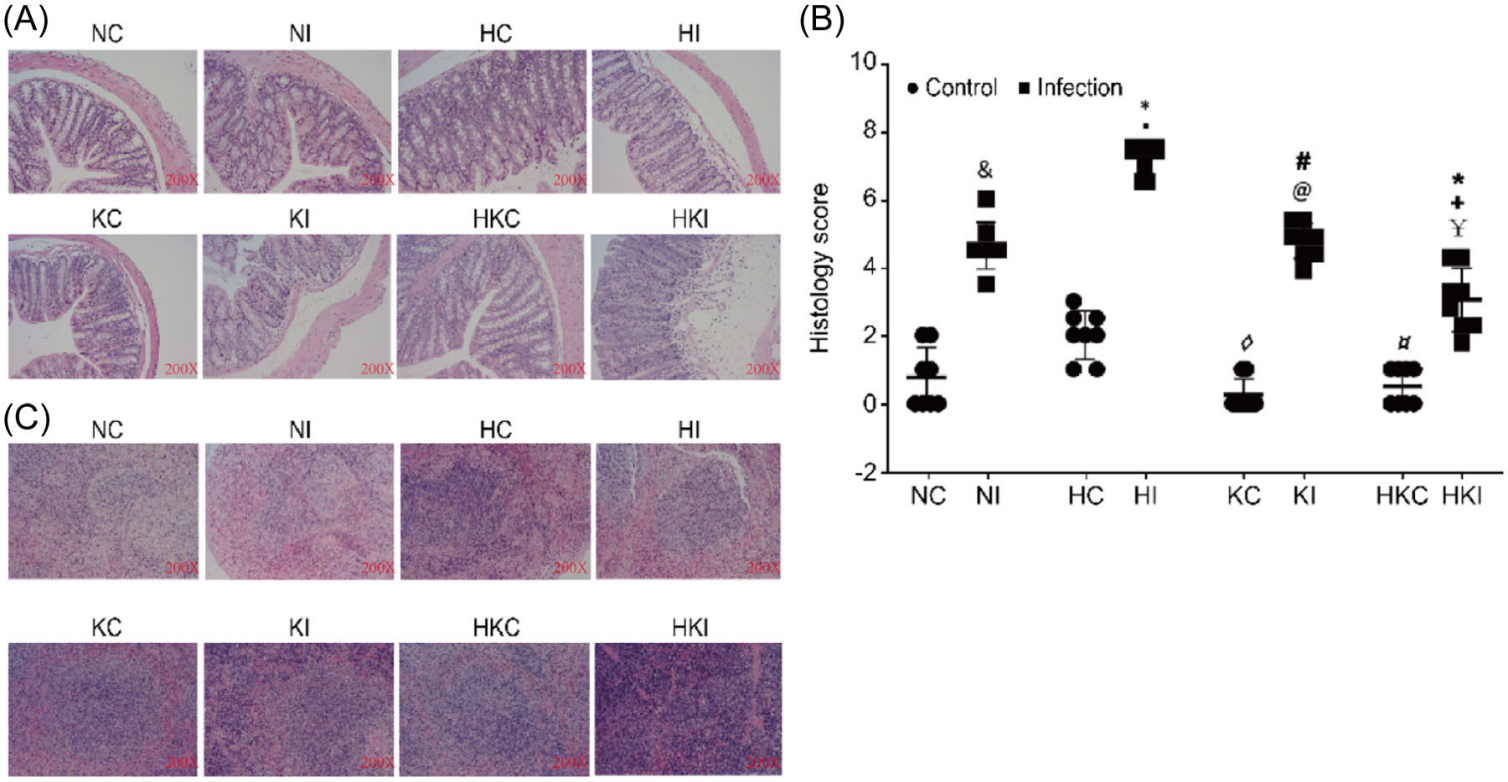
(A) Pathological sections of the colons of mice in each group (200×). (B) Results of pathological scoring of mice in each group. (C) Histopathological sections of the spleens of mice in each group (200×). The WT normoxia control group (NC), MPO^‐/‐^ normoxia control group (KC), WT *Citrobacter rodentium* infection group (NI), MPO^‐/‐^ *C. rodentium* infection group (KI), WT hypoxia control group (HC), MPO^‐/‐^ hypoxia control group (HKC), WT hypoxia C. *rodentium*‐infected group (HI) and MPO^‐/‐^ hypoxia *C. rodentium*‐infected group (HKI).

The results of H&E staining of colon tissue showed (Figure 4A) that the structure of the small intestinal mucosa of the mice in each control group was clear and complete, the villus crypts were deep, and the villi were neatly arranged. We found that the pathological damage to colon tissue was more severe in the HI and KI groups as a result of colon edema, thinning, disorganized and variable villi, inflammatory cell infiltration around crypts, and shallow crypts.

We evaluated the pathological changes in colon tissue in each group of mice. The results (Figure 4B) showed that the pathological changes of the mice in the HI group were the most serious, and the normal tissue structure of part of the mucosa was significantly damaged. Compared with the control group, the HI group had significant differences in colon pathological tissue scores after infection. However, comparing the pathological scores of the HKI group with the KI group and the HI group with the NI group, the scores were significantly lower (*p* < .05), indicating that MPO could increase the inflammatory response in mice. Figure 4C shows that the spleens of mice in the NI and KI groups had a large amount of inflammatory cells infiltration.

### 3.4 The effect of MPO in inflammatory response of *C. rodentium*‐infected mice under hypoxia

**Figure 5:**
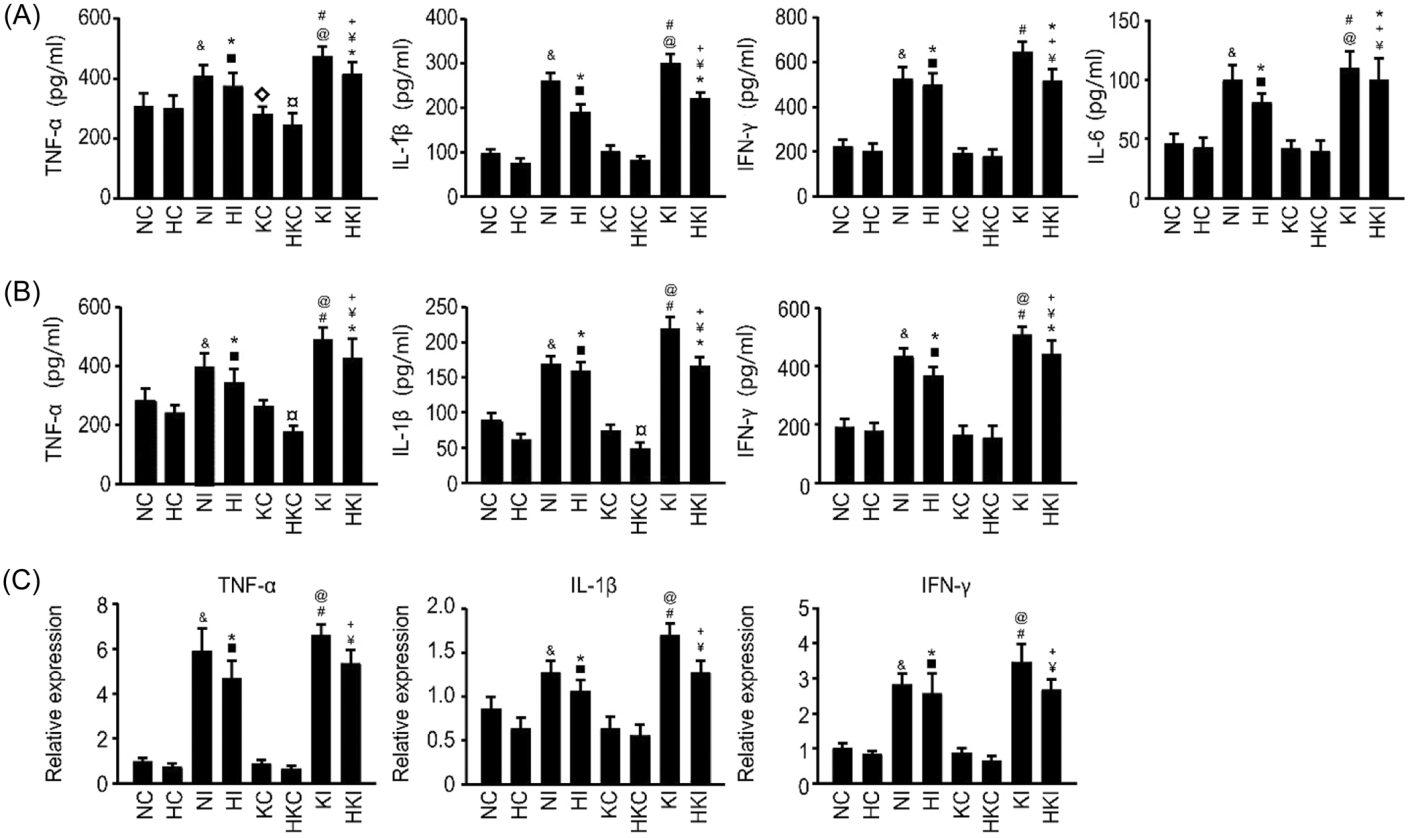
(A) Detection of related inflammatory factors in serum by ELISA. (B) Detection of related inflammatory factors in the spleen by ELISA. (C) Detection of inflammatory factors in the colon by PCR. The WT normoxia control group (NC), MPO^‐/‐^ normoxia control group (KC), WT *Citrobacter rodentium* infection group (NI), MPO^‐/‐^ *C. rodentium* infection group (KI), WT hypoxia control group (HC), MPO^‐/‐^ hypoxia control group (HKC), WT hypoxia C. *rodentium*‐infected group (HI) and MPO^‐/‐^ hypoxia *C. rodentium*‐infected group (HKI).

Compared with the control mice, the infected mice had significantly increased expression levels of the inflammatory factors TNF‐α, IFN‐γ, IL‐6, and IL‐1β in the serum, spleen, and colon (*p* < .05). Comparing WT mice, these inflammatory factors in MPO^‐/‐^ mice were significantly increased in serum, spleen, and colon tissues (*p* < .05). However, the expression of the above factors decreased under hypoxia exposure, which suggests that the immune response was downregulated under hypoxia (*p* < .05) (Figure 5A–C).

### 3.5 The effect of MPO in the chemotaxis of neutrophils and macrophages of *C. rodentium*‐infected mice under hypoxia

**Figure 6:**
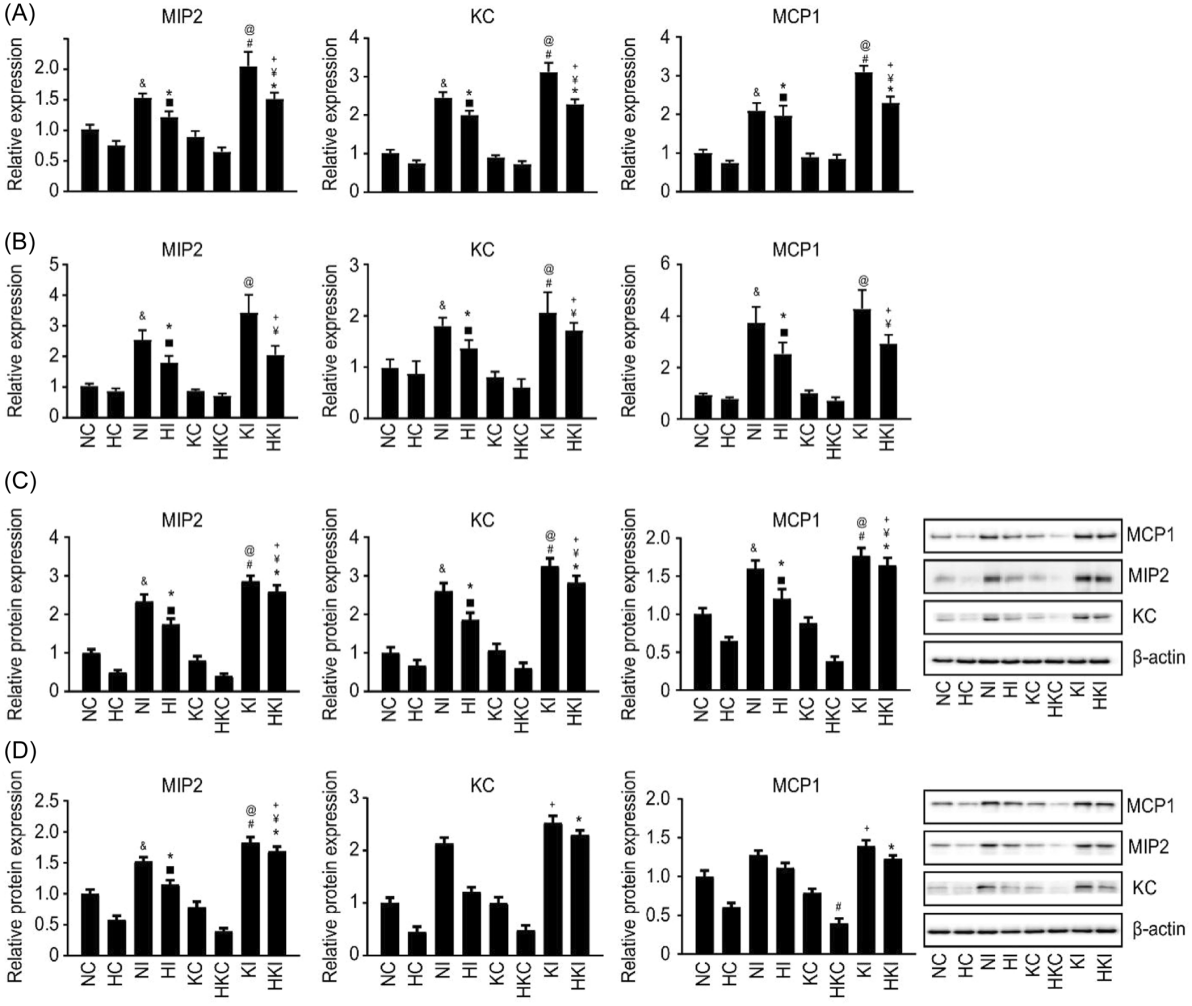
(A) Detection of phagocytic chemokines in the spleen by PCR. (B) Detection of phagocytic chemokines in the colon. (C) Detection of related chemokines in the spleen by WB. (D) Detection of related chemokines in the colon by WB. The WT normoxia control group (NC), MPO^‐/‐^ normoxia control group (KC), WT *Citrobacter rodentium* infection group (NI), MPO^‐/‐^ *C. rodentium* infection group (KI), WT hypoxia control group (HC), MPO^‐/‐^ hypoxia control group (HKC), WT hypoxia C. *rodentium*‐infected group (HI) and MPO^‐/‐^ hypoxia *C. rodentium*‐infected group (HKI).

**Figure 7:**
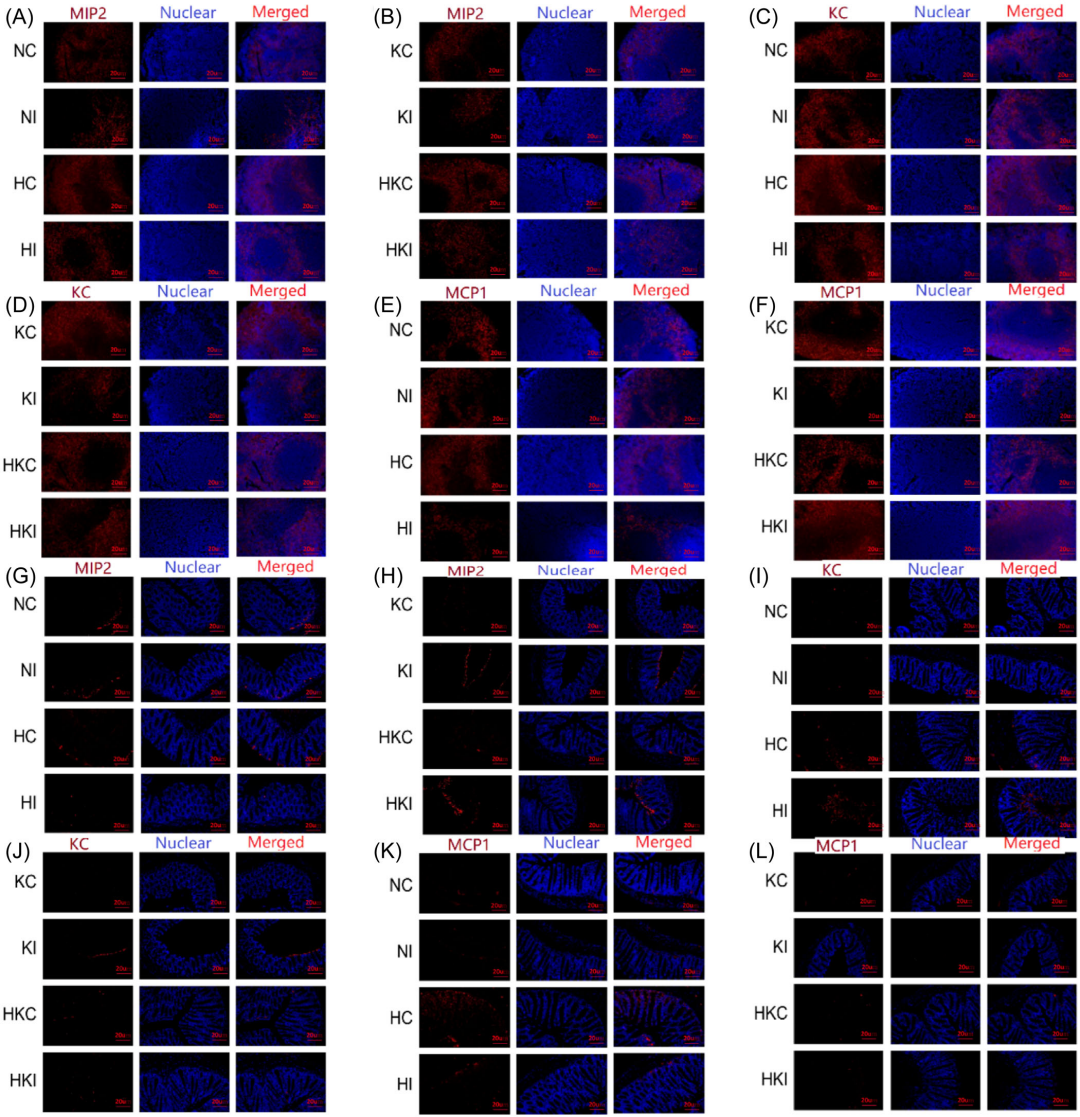
Results of immunofluorescence staining for chemokines in the spleen and colon of mice in each group. The WT normoxia control group (NC), MPO^‐/‐^ normoxia control group (KC), WT *Citrobacter rodentium* infection group (NI), MPO^‐/‐^ *C. rodentium* infection group (KI), WT hypoxia control group (HC), MPO^‐/‐^ hypoxia control group (HKC), WT hypoxia C. *rodentium*‐infected group (HI) and MPO^‐/‐^ hypoxia *C. rodentium*‐infected group (HKI). (A) MIP2 for WT‐spleen. (B) MIP2 for MPO^‐/‐^ spleen. (C) KC for WT‐spleen. (D) KC for MPO^‐/‐^ spleen. (E) MCP1 for WT‐spleen. (F) MCP1 for MPO^‐/‐^ spleen. (G) MIP2 for WT‐colon. (H) MIP2 for MPO^‐/‐^ colon. (I) KC for WT‐colon. (J) KC for MPO^‐/‐^ colon. (K) MCP1 for WT‐colon. (L) MCP1 for MPO^‐/‐^ colon.

The results showed that compared with those of the control groups (Figure 6A–D), the expression levels of MIP2, KC, and MCP1 in the spleen and colon of each infected group were significantly increased (*p* < .05), while in hypoxia, the expression levels of MIP2, KC, and MCP1 in the spleen and colon were decreased compared with those in the control condition (*p* < .05), which suggested that the neutrophil and macrophage chemotaxis capacity was reduced in hypoxia. The results were further confirmed by fluorescence analysis, which showed that compared with that in the normal groups, the expression of MIP2, KC, and MCP1 in the infected groups was significantly increased in both spleen and colon tissues, and the content of these chemokines in the MPO^‐/‐^ groups was even greater. The independent experiments were repeated three times (Figure 7A–L).

## 4 DISCUSSION

Previous studies have shown that hypoxia leads to abnormal systemic immune function, which may be closely related to the development of various chronic diseases.^10^, ^11^ The oxygen‐sensing mechanism in the body can regulate the expression of genes under exposure to different oxygen concentrations.^12^ An important cellular metabolic feature of hypoxia is the elevated expression of hypoxia‐inducible factor (HIF‐1), which participates in human physiological and pathological processes by regulating a variety of related genes.^13^ Experimental colitis caused by *C. rodentium* can produce a large amount of pro‐inflammatory cytokines and severely damage the layer of epithelial cells.^14^, ^15^ The levels of the pro‐inflammatory cytokines TNF‐α, IL‐6, and IL‐1β in colon tissues are significantly increased with the occurrence of IBD.^16^, ^17^

MPO can participate in the regulation of various inflammatory responses and pathological effects in the body, Swaminathan et al. found that fecal MPO is an accurate biomarker of endoscopic activity in IBD and predicted a more complicated IBD course during follow‐up.^18^ So we focused on the changes in MPO levels on the pathological response of *C. rodentium*‐induced colitis in mice in a hypoxic environment. After infecting WT mice and MPO‐/‐ mice with C. *rodentium*, we found that MPO‐/‐ mice excreted more bacteria and lost more weight. This is mainly because neutrophils in MPO‐/‐ mice cannot function to clear bacteria normally in the absence of MPO. Subsequently, we further analyzed the results and found that the pathological changes of C. *rodentium*‐induced enteritis in MPO‐/‐ mice in a normoxic environment were more severe, mainly manifested as obvious submucosal edema and a large amount of neutrophil and macrophage infiltration. Goblet cell numbers decreased significantly, and intestinal epithelial integrity was significantly damaged. A large number of inflammatory factors and chemokines were produced in large quantities with the occurrence and development of infection.

However, the intestinal inflammatory pathological damage was relatively mild in hypoxic mice compared with that in WT C. *rodentium*‐induced enteritis in MPO‐/‐ mice. The colon length was shortened and inflammatory response was increased in C. *rodentium*‐mice with a normoxic environment, which was all alleviative under hypoxic exposure. We supposed that a hypoxic environment can reduce the innate immune function of mice and then reduce the inflammatory response. Neutrophils mainly rely on respiratory oxidative bursts^19^ and the production of reactive oxygen species^20^ to destroy pathogens. However, hypoxia causes insufficient oxygen pressure in the environment, resulting in insufficient recruitment of NOX2 in neutrophils and insufficient production of reactive oxygen species, which can lead to a decrease in the ability to clear pathogens.

Macrophages are the main phagocytic cells in the intestine and are closely related to the pathogenesis of IBD.^21^ In the acute phase, the number of macrophages significantly increased in the intestinal mucosa in IBD. In our study, it was found that although a hypoxic environment can increase the chemotaxis of macrophages, macrophage activation is reduced due to the lack of oxygen. HIF‐1, a heterodimeric transcription factor, played a key role in the adaptation of macrophages to changes in oxygen tension, and HIF‐1α, as the active subunit of HIF‐1, was the main functional factor. After macrophages were activated, it adapted to the stimulation of inflammatory factors through the transformation of metabolic pathways. The hypoxic environment in our experiment significantly upregulated HIF‐1α expression, which resulted in an increase in the glycolysis pathway.^22^

In conclusion, in the *C. rodentium*‐induced colitis mouse model, MPO can not only affect bacterial clearance but also participate in pathological damage to tissues. A hypoxic environment can reduce the innate immune function of mice. Targeting MPO and hypoxic environment in the future may mitigate oxidative damage to host tissue and ensuing inflammation so as to protect the colon from inflammatory injury.

## AUTHOR CONTRIBUTIONS

Wei Zhang contributed to the research design. Xiang Gao performed most of the experimental work, acquired and analyzed the results, and wrote the first draft of the manuscript. Qingfang Zhu, Ying Han, Ruhan Jia, Yu Zhang, and Xiang Gao performed the experimental work and analyzed the results. Wei Zhang and Yu Zhang designed, supervised, and interpreted the experimental data. Wei Zhang critically revised the manuscript. Xiang Gao and Wei Zhang wrote the final version of the manuscript. All listed authors agreed to be the author.

## CONFLICT OF INTEREST STATEMENT

The authors declare no conflict of interest.

## ETHICS STATEMENT

This study was approved by the Ethics Committee of the Medical College of Qinghai University.

## Supporting information

Supporting information.

## Data Availability

The data that support the findings of this study are available from the corresponding author upon reasonable request.
